# Exploring the Establishment of Hospice Service System Integrating Medical Care and Funeral Services

**DOI:** 10.34172/ijhpm.2022.7606

**Published:** 2023-01-01

**Authors:** Yunling Wang, Weijia Tan, Yifang Wang

**Affiliations:** ^1^Department of Medical Psychology and Ethics, School of Basic Medical Sciences, Shandong University, Jinan, China.; ^2^School of Marxism, Shandong First Medical University (Shandong Academy of Medical Sciences), Tai’an, China.; ^3^Factor-Inwentash Faculty of Social Work, University of Toronto, Toronto, ON, Canada.; ^4^The School of Health Humanities, Peking University, Beijing, China.

## Introduction: The Separate Management of Life and Death

 In terms of the development of hospice, 1988 is the first year of the establishment of hospice in mainland China. In 1988, with the initiative and funding of Chinese-American Professor Tianzhong Huang, Tianjin Medical College (now Tianjin Medical University) set up the Tianjin Hospice Center.^1^ In October 1998, the Shanghai Retired Workers Nanhui Nursing Home was the first hospital in mainland China to provide in-patient care for retired workers who were terminally ill.^1^ In October 2017, the National Health Commission designated five pilot cities (Beijing, Changchun, Shanghai, Luoyang, and Deyang) to explore different types of hospice services, strategies of service delivery, and modes of finances.^2^ The five pilot cities are located in different parts of China, and among them, there is great variety in population size, economy, educational level, and social development, which means their exploration can be representative and generalized to other parts of China. In May 2019, the second batch of pilot cities, including 71 cities across China, were launched, accelerating the establishment of China’s hospice service system.^2^ This article is a proposal for a changed relationship between hospice and funeral services, one which is being piloted in China and for which data will be available in 2023.

 The policy of promoting hospice aims at the important goal of improving the quality of death. Generally speaking, the quality of death refers to a comprehensive evaluation of the end-of-life quality of patients and their experience and feelings during the dying process.^3,4^ Likewise, the establishment of China’s hospice system aims at improving the quality of death for its citizens. In Chinese society, life and death are important matters, so the handling of life and death is often not only about the technical level of medical care, but also related to non-technical aspects, such as social work, psychological care, grief counseling, and so forth.^5^ These non-technical aspects are intertwined with funeral culture. After the concept of hospice, which originated in Western society and culture, was introduced to China, people strove to learn end-of-life care techniques.^5^ However, culturally, people were challenged to enter into necessary conversations around death, the handling of life and death, and the understanding of filial piety.^5,6^ The separate management of life and death that originated from modern Western culture has become a dilemma facing the modern hospice system in the context of Chinese culture.^6,7^

 Then what is the dilemma? In a traditional Chinese agricultural society, where the economy and medicine are underdeveloped, the scene of death is usually like this: the dying are moved to the central room by their children and are accompanied by the children; other relatives are on the periphery, and the dying are in a family setting; after the passing away, the children bathe the deceased and change their clothes.^8^ In some parts of China, people would bathe, groom, and change clothes for the dying before the death, lest old people pass away naked.^9^ To ease the pain of bereavement, immediate family are often assisted by relatives and friends to bury the deceased in accordance with cultural customs. Studies have shown that in modern cities where there are few traditional funeral custom, patients with mental disorders caused by the inability to survive the grief after the death of a loved one, account for 15% to 21% of mental health outpatients.^10^ Whereas in rural areas where psychological counseling and psychotherapy are not yet widespread, the bereaved are usually able to withstand the heavy blow of losing loved ones and are more likely to adapt to the changes brought about by the deaths of their beloved ones.^10^ It is believed that traditional funeral customs serve as psychotherapeutic group counseling.

 However, under the background of industrialization, human death in China is becoming more and more rigid and inhuman. Kübler-Ross argues that, because patients are often forced to leave their familiar environment and are rushed to the emergency room, the process of death becomes lonely and unsympathetic.^11^ Fu asserts that, in the modern industrial society, terminally ill patients are often sent directly from the hospital to the funeral home instead of returning to their homes after they pass away.^12^ He deems this way of handling dead bodies as the inevitable result of industrialization, urbanization, and smaller family size.^12^ After a simple ceremony, the body is sent to the crematorium and then to the cemetery.^13^ Such a brief funeral process makes it difficult for the bereaved to concentrate on their grief, and the help of relatives and friends is redundant, as it is no longer possible for them to help. Everything is fast and efficient, and the whole funeral process has been declared over before verbal comforting and psychological companionship take place.

 On the issue of the rare human touch at the end of life, hospice can effectively change the problem of over-intervention from modern medicine in the process of human death, thus changing what Kübler-Ross called the process of death becoming lonely and inhuman.^11^ However, the design of the existing hospice system in China cannot change the inhuman condition described by Fu,^12^ because it cannot change the current state of the separate management of life and death. The phenomenon of separate management of life and death refers to that hospitals overseeing patients’ medical treatment and care while funeral homes take the responsibility to deal with the affairs related to the deceased. The two sectors are not connected and are independent departments from each other, which causes people to struggle between the two departments before and after the death of the patient. This mode of bereavement is quite different from traditional Chinese society, which relies on the help of relatives, friends, and neighbors, and follows traditional funeral customs. Relatives, friends, and neighbors are people who understand the stories of the deceased as well as the pain of the bereaved with compassion for the deceased and bereavement so that they can provide the bereaved with psychological and spiritual care. Modern funeral home staff are strangers to the bereaved, as they do not understand either the story of the deceased or what the bereaved is suffering, so it is difficult for the staff to show sentiments in the funeral home setting. Even if they have a strong sense of humanity and show support and encouragement to the bereaved, it is difficult for the staff to quickly establish a necessary trust relationship needed in interpersonal communication, and the bereaved cannot have a true emotional resonance with the staff’s support and encouragement. It is also frustrating for the bereaved to get psychological support and spiritual care from funeral home staff, so as to relieve their inner grief. What’s more, because it is usually challenging for the bereaved to make rational judgments and decisions in great pain, if someone uses the opportunity of bereavement to blackmail or extort money from them, the bereaved may suffer additional financial losses, which adds to their misfortune.^14^ This is the real dilemma caused by the separate management of life and death.

## The Establishment of Hospice Service System Integrating Medical Care and Funeral Service

 We argue that hospice is not only supposed to alleviate the excessive intervention of modern medicine in the process of death; it should also break through the dilemma caused by the separate management of life and death. Therefore, it is imperative to establish a system of hospice service integrating medical care and funeral affairs.

 Specifically, we propose that funeral service professionals can become involved in hospice work for terminally ill patients. Funeral service professionals may, subject to conditions, join the hospice team, get involved in clinical deaths in advance, participate in the accompanying, caring, and comforting of terminally ill patients, listen to their life stories, and observe their treatment. Funeral service professionals can experience the value of collaboration through communication with other members of the team. They can experience the hardships of caregivers through communication with family members; they can understand the meaning of life by listening to patients’ stories; they can understand the value of medical care by observing the work of medical professionals. In this process of early involvement, funeral service professionals can enrich their understanding of life and living, and knowledge about medical care, nursing, social work, and hospice. Thus, they can construct their own values of life care and appreciate and even reconstruct their professional values. Most importantly, through this early involvement, funeral service professionals can develop the ability to share the life and family stories of terminally ill patients by switching from strangers to acquaintances who are caring for patients and being accepted by patients and their families. This ability to show empathy is the prerequisite for funeral service professionals to be able to serve the bereaved with wholehearted efforts and genuine sentiments.

 On the other hand, we argue that medical professionals can extend their services past the death of the patient, involving themselves in the funeral as well. The hospice care team can extend their services for the clinical death of the patient, participate in the management of the biological death, and assist in the funeral affairs of the deceased. As helpers or acquaintances, medical professionals can help arrange the disposal of the body, plan funerals, and attend memorials, bidding farewell to and mourning the deceased. Their participation can help create a space for the bereaved to vent emotions; it can also help medical professionals savor the value of mourning, enrich personal experience, and offer grief care to the bereaved. Such institutional arrangements avoid the situation in which medical professionals discontinue their services and care at the end of the patient’s life but extend them to the subsequent funeral process.

## Implications of the Establishment of an Integrated Hospice Service System

 It is an important proposition to transform the separate management of medical services and funeral affairs and to realize the integration of medical care and funeral services for the establishment of a hospice service system. First, for the dying, the integrated hospice service system can help them receive holistic care services from living to death near the end of their life and achieve both physical and mental closure with little body pain or psychological regret. A hospice system that is not incorporated into the funeral service focuses on making sure that the patients suffer no pain. Although mental and spiritual care would be involved, the separate hospice system focuses largely on non-funeral matters. In fact, in the case of the separate management of life and death, the staff of the hospice team does not have the capacity to deal with funeral affairs, let alone make a commitment to the dying. However, studies have shown that arranging funeral matters is the last wish that dying people often express, such as the funeral ceremony, disposal of the remains, distribution of bequests, even the guests to be invited to the funeral, and so forth.^15,16^ In the integrated hospice service system, funeral staff who join the hospice team can not only answer questions from the dying about funeral affairs in a professional manner but also make solemn commitments, so as to meet the needs of the dying in a more complete manner and help realize their wish to pass away with little regret.

 Second, for the bereaved, the funeral staff of the integrated hospice service system can help relieve them of worries, which is conducive to alleviating the pain of bereavement. For the bereaved who have lost their beloved one, great grief often makes it difficult for them to deal with their own lives properly. What’s more, mourning becomes a process rather than a state,^14,17^ while staff from an interdisciplinary and interprofessional hospice team can work together to arrange the funeral affairs of the deceased properly and thoughtfully. Funeral service staff stand a good chance of becoming “familiar strangers,” relatively speaking, to patients and their families over a longer period of time, or becoming like their close friends. The funeral service staff, thus, have empathy with patients and their families, so that they can gain their trust, and invest more energy and emotions at work. Such an integrated hospice service system can basically replicate the kind of bereavement mechanism that exists in the traditional agricultural society, which means the bereaved, with the help of relatives, friends, and neighbors, bury the deceased properly into the soil in accordance with traditional customs and have their grief relieved in the process as well. Under this mechanism, the bereaved can be spared the complicated process of dealing with funeral affairs, so that they can focus on receiving grief support and ultimately have their bereavement pain alleviated. As a result, the goal of a peaceful handling for the bereaved and the deceased of hospice can be achieved.

 Finally, for society, the construction of an integrated system also has an essential significance; that is, when both parties, from the medical care and the funeral service, striving for common professional values, work together to provide humane care at the end of human life, they can avoid value disorientation, misleading behaviors, and disputes with the bereaved due to the transfer between medical care and funeral services. They can also avoid the lurking perils of the doctor-patient relationship as well as the relationship between the funeral staff and the bereaved. To be more explicit, in the scenario of separate management of life and death, although medical staff have a sense of sanctity and awe for life, all of these stop at the moment when terminally ill patients stop breathing. Medical practitioners no longer spend time and energy on a lifeless person, and the value of their profession cannot be reflected through a lifeless person. For funeral staff, their professional values seem to lie more in the delicate make-up of the body, the reasonable and subtle arrangement of the ceremony, and other services to the bereaved, not reflected through the awe, compassion, and pity for life. This different understanding of their respective professional values can easily lead to value disorientation for medical and funeral professionals. For medical practitioners, the deceased with no medical value is no longer the object of their work, and therefore there is no need to put more energy into the person. For funeral workers, the deceased is only a body; their service is for the bereaved, while the matters related to the deceased are only the content of their work. Hence, both sides of the medical and funeral staff may find it difficult to show due to awe and compassion for the deceased who used to be a living person, where it is easy to lack the human touch. Whereas in an integrated hospice service system, both medical and funeral professionals share a unified professional value, that is, out of respect for life and human nature, they give thoughtful humane care to the deceased and their families.

 Nonetheless, challenges are also identified. The first is about staffing. Doctors are costly and are pressed for time; it would not be feasible for them to care for the dead.^15^ Additionally, medical professionals and funeral staff receive different training on treating patients and interacting with families.^15^ We argue that professional hospice workers should be trained in China. Professional hospice workers should receive training on basic medical and nursing knowledge, death education, and grief support. They will be the main staff to assist with both before- and after-death affairs. Nevertheless, education on hospice can still be provided for medical and funeral service students and staff, and they can be involved as volunteers. Also, we believe a well-equipped hospice service team should be a multidisciplinary professional team; therefore, the roles of other related professionals can be explored as well. For instance, in some cases of the pilot cities in China, medical professionals, social workers, and volunteers from healthcare facilities and funeral homes have worked together to ensure patients go through the end of life as comfortably and painlessly as possible. During the process, nurses provide medication to manage pain and discomfort; support staff provide specialized services, such as massage and music therapy; social workers and psychotherapists provide counseling to patients and families in need; and volunteers take on various roles, including chatting with patients, helping them realize end-of-life wishes, and helping family members prepare for transits.^18^ The first author of this article has interviewed medical and funeral service professionals from several pilot cities, and the first and second authors are working on the data analysis. We hope to understand the experience of the front-line practitioners and provide more practical recommendations on hospice staffing and training.

 The other challenge is payment. So far, there has been no unified payment system as all pilot cities are exploring individual ones. Most of the pilot cities adopt a Diagnosis Related Group payment method.^18^ A Diagnosis Related Group payment method is based on the combination of cases, drawing on big data, taking into consideration the individual characteristics of the cases, such as age, gender, length of hospital stay, clinical diagnosis, disease, surgery, disease severity, comorbidities, complications, outcomes, and other factors.^19^ It is a system that divides cases with similar clinical processes and costs into the same group for management, and formulates medical cost standards for payment on a group-by-group basis.^19^ However, since the individual needs for hospice services are diverse, and services such as psychotherapy, counseling, and grief support are currently not included in any existing groups, a payment system that is suitable for Chinese society should be further explored. We suggest promoting a gradual incorporation of home and institutional hospice services into the existing basic medical insurance, long-term care insurance, and other supplementary medical insurance in China. Government-purchased services are another way to support the promotion of hospice services. The first author of this article has distributed questionnaires and surveys about payment systems in pilot cities and hopes to provide relevant policy recommendations based on quantitative studies in the future.

 To conclude, the establishment of the integrated hospice service system is essentially a new exploration and expansion of the concept and practice model of hospice. Handling funeral affairs has a significant bearing on both the dying and the bereaved, which contains important humanistic connotations. Including funeral staff in the hospice system as an important subject for practice can in many cases enable them to play roles “similar to relatives” and “on behalf of relatives,” roles which embody the humanistic care for the deceased. An integrated hospice service system can effectively provide psychological and emotional support, and strengthen the practical role of multiple subjects in the hospice,^20^ which is an innovative measure in the construction of the hospice system.

## Ethical issues

 Not applicable.

## Competing interests

 Authors declare that they have no competing interests.

## Authors’ contributions

 YuW: Drafting of the manuscript and obtaining funding. WT: Drafting of the manuscript. YiW: Conception and design, critical revision of the manuscript for important intellectual content, and supervision.

## Funding

 This work was supported by the National Social Science Fund of China project “Study of the construction of hospice service system with Chinese characteristics against the strategic background of Healthy China” [grant number: 19BSH187].

## Disclaimer

 The views expressed in the article are the authors’ own and not an official position of the institutions or funder.
